# Qinhuo Shanggan oral solution resolves acute lung injury by down-regulating TLR4/NF-*κ*B signaling cascade and inhibiting NLRP3 inflammasome activation

**DOI:** 10.3389/fimmu.2023.1285550

**Published:** 2023-10-25

**Authors:** Shun Tang, Yuanjing Liang, Minmin Wang, Jiarong Lei, Yuhui Peng, Qiu Tao, Tianqi Ming, Wenyu Yang, Chuantao Zhang, Jinlin Guo, Haibo Xu

**Affiliations:** ^1^ State Key Laboratory of Southwestern Chinese Medicine Resources, Department of Pharmacology, School of Pharmaceutical Sciences, Chengdu University of Traditional Chinese Medicine, Chengdu, China; ^2^ School of Food and Bioengineering, Xihua University, Chengdu, China; ^3^ Department of Respiratory Medicine, Hospital of Chengdu University of Traditional Chinese Medicine, Chengdu, China; ^4^ State Key Laboratory of Southwestern Chinese Medicine Resources, School of Medical Technology, Chengdu University of Traditional Chinese Medicine, Chengdu, China

**Keywords:** acute lung injury (ALI), TLR4 (toll-like receptor 4), NF- kappa B, NLRP3, cytokine

## Abstract

Acute lung injury (ALI) is a common condition, particularly in the COVID-19 pandemic, which is distinguished by sudden onset of respiratory insufficiency with tachypnea, oxygen-refractory cyanosis, reduced lung compliance and diffuse infiltration of pulmonary alveoli. It is well-established that increasing activity of toll-like receptor 4 (TLR4)/nuclear factor kappa-B (NF-*κ*B) signaling axis and the NOD-, LRR- and pyrin domain-containing protein 3 (NLRP3) inflammasome activation are associated with the pathogenesis of ALI. Since ALI poses a huge challenge to human health, it is urgent to tackle this affliction with therapeutic intervention. Qinhuo Shanggan oral solution (QHSG), a traditional Chinese herbal formula, is clinically used for effective medication of various lung diseases including ALI, with the action mechanism obscure. In the present study, with the rat model of lipopolysaccharide (LPS)-induced ALI, QHSG was unveiled to ameliorate ALI by alleviating the pathological features, reversing the alteration in white blood cell profile and impeding the production of inflammatory cytokines through down-regulation of TLR4/NF-*κ*B signaling cascade and inhibition of NLRP3 inflammasome activation. In LPS-stimulated RAW264.7 mouse macrophages, QHSG was discovered to hinder the generation of inflammatory cytokines by lessening TLR4/NF-*κ*B signaling pathway activity and weakening NLRP3 inflammasome activation. Taken together, QHSG may resolve acute lung injury, attributed to its anti-inflammation and immunoregulation by attenuation of TLR4/NF-*κ*B signaling cascade and inhibition of NLRP3 inflammasome activation. Our findings provide a novel insight into the action mechanism of QHSG and lay a mechanistic foundation for therapeutic intervention in acute lung injury with QHSG in clinical practice.

## 1 Introduction

Acute lung injury (ALI) is an acute inflammatory condition often triggered by severe infection or trauma. It is characterized by difficulty in breathing, respiratory distress, and damage to the alveolar epithelial barrier (1, 2). The primary pathological features include acute widespread alveolar damage, pulmonary interstitial fluid accumulation, heightened pulmonary capillary permeability, and excessive inflammation (3, 4). Unfortunately, the onset of ALI is typically sudden and rapidly progressing, and it bears a grim prognosis (5, 6). It can potentially lead to failures in the respiratory and circulatory systems, acute respiratory distress syndrome (ARDS) and, in severe cases, even death. Amid the COVID-19 pandemic, the ALI/ARDS mortality rate stands at a staggering 40% (7), which significantly perturbs the quality of life of the patients.

Lipopolysaccharide (LPS), a principal part of the cytoderm of gram-negative bacteria, activates the pathogen-associated molecular pattern (PAMP), which is a primary cause of ALI prompted by gram-negative bacteria infection (8). With the help of proteins such as LPS-binding protein, myeloid differentiation protein 2 (MD2), and cluster of differentiation protein 14 (CD14), LPS binds to the toll-like receptor 4 (TLR4) and activates the nuclear factor kappa-B (NF-*κ*B) signaling pathway, provoking a pro-inflammatory reaction (9), which results in the release of inflammatory cytokines like tumor necrosis factor-alpha (TNF-α), interleukin-6 (IL-6), interleukin-1beta (IL-1β) and interferon-gamma (IFN-γ), triggering and amplifying inflammatory responses (10). Accumulating research indicates that inhibition of TLR4/NF-*κ*B signaling pathway can essentially reduce lung tissue injury and hinder the transduction of inflammatory signals, suggesting its integral role in mediating LPS-caused ALI (11–13).

Moreover, the activation of the NOD-, LRR- and pyrin domain-containing protein 3 (NLRP3) inflammasome is strongly associated with various lung disorders such as ALI, chronic obstructive pneumonia, asthma, and pulmonary fibrosis (14). NLRP3 inflammasome is composed of three components including a sensor protein NLRP3, an adaptor ASC (apoptosis-associated speck-like protein containing a caspase recruitment domain, also named Pycard) and an effector pro-caspase-1 (the precursor molecule of caspase-1) (15). Upon activation by LPS, NLRP3 inflammasome triggers the formation of active caspase-1 (CASP1). Then, caspase-1 proteolytically cleaves pro-IL-1β at Asp116 into IL-1β, pro-IL-18 into IL-18, and gasdermin D (GSDMD) into C-terminal domain (GSDMD-C) and N-terminal domain (GSDMD-N). The GSDMD-N forms some pores on the cytoplasmic membrane to facilitate the secretion of inflammatory cytokines IL-1β and IL-18, and the induction of pyroptosis, a pro-inflammatory form of programmed cell death (16).

Traditional Chinese medicine (TCM) may possess some advantages in the treatment of various diseases including ALI, due to the diverse bioactive natural components, minimal toxicities, and limited side effects (17–19). Research has demonstrated that TCM exhibits the ability to alleviate lung injury through anti-inflammation, anti-oxidant stress response and safeguarding the integrity of airway epithelial barriers (20, 21). Qinhuo Shanggan oral solution (QHSG), a Chinese herbal formula containing *Scutellariae radix*, *Pogostemonis herba*, *Armeniacae semen amarum*, *Ephedrae herba*, *Magnoliae flos*, *Angelicae dahuricae radix*, *Platycodonis radix* and *Belamcandae rhizoma*, is used in the clinical setting for the medication of various lung diseases, including ALI. QHSG exhibits properties of ameliorating ALI-related symptoms, reducing inflammation, relieving cough, alleviating asthma and expelling phlegm. However, the molecular mechanism responsible for the efficacy of QHSG on ALI remains unknown. Therefore, we sought to investigate the effect of QHSG on ALI and unravel the action mechanism of QHSG in the context of TLR4/NF-*κ*B signaling pathway and NLRP3 inflammasome, laying a foundation for the wider application of QHSG in clinical practice.

## 2 Materials and methods

### 2.1 Chemicals and reagents

Qinhuo Shanggan oral solution (QHSG), a Chinese herbal formula containing *Scutellariae radix*, *Pogostemonis herba*, *Armeniacae semen amarum*, *Ephedrae herba*, *Magnoliae flos*, *Angelicae dahuricae radix*, *Platycodonis radix* and *Belamcandae rhizoma* was supplied by the Affiliated Hospital of Chengdu University of Traditional Chinese Medicine. Lipopolysaccharide (LPS) (#L2880) was purchased from Sigma-Aldrich (USA). Antibodies to TLR4 (#AF7017), NF-*κ*B p65 (#AF5006), phospho-NF-*κ*B p65 (Ser276) (#AF3387), recombinant inhibitory subunit of NF-*κ*B alpha (I*κ*Bα) (#AF5002), NLRP3 (#DF7438), IL-6 (#DF6087), and IFN-γ (#DF60445) were acquired from Affinity Biosciences (Jiangsu, China). Antibody against ASC (#A22046) was bought from ABclonal Technology (Wuhan, China). Primary antibodies to GSDMD (#20770-1-AP), CASP1 (#22915-1-AP), IL-18 (#10663-1-AP), IL-1β (#26048-1-AP), TNF-α (#26405-1-AP), and secondary antibodies were gotten from Proteintech (Wuhan, China). Rat tumor necrosis factor-α (TNF-α) ELISA kit (#HEA133Ra) and rat interleukin-1β (IL-1β) ELISA kit (#HEA563Ra) were provided by Cloud-Clone Corporation (Wuhan, China). Dexamethasone (DEX) was obtained from Keen Experimental Equipment Co. (Chengdu, China). All other reagents were readily available commercially.

### 2.2 Preparation of QHSG

QHSG consisting of 8 herbs (**Table 1**) was manufactured and clinically utilized by the Affiliated Hospital of Chengdu University of Traditional Chinese Medicine. Briefly, *Armeniacae semen amarum* was boiled in 5 times the amount of water for 30 min twice, and the aqueous extracts were collected and mixed. After the *Magnoliae flos*, *Angelicae dahuricae radix* and *Pogostemonis herba* were boiled to yield the distillate, the aqueous extract was harvested, and the solid residue was boiled with *Scutellariae radix*, *Ephedrae herba*, *Platycodonis radix* and *Belamcandae rhizoma* in 10 times the amount of water for 60 min thrice. All the aqueous extracts were mixed, filtered and condensed, followed by addition of above distillate, which made the QHSG. For *in vitro* experiments, QHSG was further concentrated in a rotary evaporator with a 60 °C-temperature water bath at a rotation speed of 2500 rpm. Eventually, the concentrate was dissolved with the cell culture medium.

**Table 1:** QHSG-contained Chinese herbs.

Chinese Name	Scientific Name	Used Part	Dosage (g)
Ma Huang	*Ephedra sinica* Stapf	dried grass stems	10
Ku Xing Ren	*Prunus armeniaca* L. var. *ansu* Maxim.	dried ripe seeds	15
Xin Yi	*Magnolia biondii* Pamp.	dried flower buds	10
Bai Zhi	*Angelica dahurica* (Fisch. ex Hoffm.) Benth. et Hook. f.	dried roots	15
Jie Geng	*Platycodon grandiflorum* (Jacq.) A.DC.	dried roots	15
She Gan	*Belamcanda chinensis* (L.) DC.	dried rhizome	15
Huang Qin	*Scutellaria baicalensis* Georgi	dried roots	15
Guang Huo Xiang	*Pogostemon cablin* (Blanco) Benth.	dried aerial part	15

### 2.3 Analysis of QHSG by high-performance liquid chromatography

For sample preparation, 2.0 ml of QHSG was diluted with 8.0 ml of methanol, and filtered with 0.22 μm-pore membranes. The standard substances including baicalin (#110715-202223), ephedrine hydrochloride (#171241-201809) and amygdalin (#110820-202109) obtained from the National Institutes for Food and Drug Control of China, were dissolved in methanol to make a solution at a concentration of 0.1 mg/ml, followed by filtration with 0.22 μm-pore membranes.

For identification of baicalin, Zorbax Eclipse Plus C18 (4.6 mm × 250 mm, 5 μm) column was used, and the gradient elution was carried out with methanol and 0.2% phosphoric acid solution (47:53) at a flow rate of 1.0 mL/min and 40 °C of column temperature. To establish the HPLC profiles of baicalin standard and QHSG, 20 μL of samples were loaded and detected by Agilent 1260 HPLC instrument at a wavelength of 280 nm.

For identification of ephedrine hydrochloride and amygdalin, Zorbax Eclipse Plus C18 column (4.6 mm × 250 mm, 5 μm) was used, with gradient elutions of methanol-0.2% phosphoric acid-triethylamine (10:90:0.2) at a flow rate of 1.0 mL/min and 40 °C of column temperature. The loading volume was 20 μL and the detection wavelength was 210 nm. The Agilent 1260 HPLC instrument was used to detect the HPLC profiles of ephedrine hydrochloride standard, amygdalin standard and QHSG, respectively.

### 2.4 Animal model and treatment regimens

The animal experimental project was licensed by the Animal Ethics Committee of Chengdu University of Traditional Chinese Medicine (Ethical Number. 2021-31). All animal experiments were in line with the Guide for the Care and Use of Laboratory Animals by International Committees. The eight-week-old SD rats weighing 200 ± 20 g each were supplied by SiPeiFu Biotechnology (Beijing, China), and housed in the standard animal facility with 12h light – 12h dark cycle, fed with standard diet and distilled water *ad libitum*. After 1 week of acclimation, the rats were randomly arranged into 6 groups: the control (C), the model (M), low-dose QHSG (5.5 g/kg), medium-dose QHSG (11 g/kg), high-dose QHSG (22 g/kg) and dexamethasone (DEX, 2 mg/kg). After anesthetization with inhalation of isoflurane, LPS at 5 mg/kg was intranasally instilled in all rats, except the control group receiving natural saline (22). Then, the rats were intragastrically administered QHSG at 5.5 g/kg, 11 g/kg and 22 g/kg or dexamethasone at 2 mg/kg respectively for 1 week, while the control and the model groups received the volume-matched vehicle. The dose selection of QHSG in this study was on the basis of its clinical dosage, and the medium-dose QHSG for rats was equivalent to that for human patients in clinical practice, by normalization with body surface area. The low-dosage and high-dosage of QHSG and the dosage of dexamethasone were chosen based on the pilot experiment (data not shown). Following the sacrifice of the rats, the organs including lung, spleen and thymus were retrieved for the following experiments.

### 2.5 Blood tests

The rats were placed in a restrainer on a rechargeable warm pad under a heat lamp to keep the rats warm. Then ventral artery sampling was conducted by drawing blood from the ventral tail artery with a needle. The white blood cell (WBC) count, neutrophil count, lymphocyte count and monocyte count were carried out on an automated hematology analyzer.

### 2.6 Organ index

Upon collection, the organs including lung, spleen and thymus were weighed. The organ index was calculated according to the formula: Organ index = Organ weight (g)/Body weight (g) × 1000.

### 2.7 Hematoxylin and eosin staining analysis

H&E staining was carried out according to the standard procedure. In Brief, the lung tissues were harvested from sacrificed rats, fixed in 4% of paraformaldehyde solution, paraffin-embedded and sliced into 5 μm-thick sections. After dewaxing with xylene and hydration with alcohol at descending concentrations and tap water, hematoxylin nuclear stain was applied to the sections, followed by thorough “blueing” of nuclei with weakly alkaline solution. After counterstaining with esoin solution, the sections were dehydrated with alcohol at ascending concentrations, rinsed in xylene and mounted, followed by blinded histopathologic evaluation under a light microscope.

### 2.8 Flow cytometry

Briefly, following the lysis of erythrocytes with lysis buffer, the peripheral heparin-anticoagulated blood T cells were stained with fluorescein isothiocyanate (FITC)-conjugated CD4 antibody (#E-AB-F1097C) (1:200), allophycocyanin (APC)-conjugated CD25 antibody (#E-AB-F1102E) (1:200) and phycoerythrin (PE)-conjugated Foxp3 antibody (12-5773-82) (1:200) (Elabscience, Wuhan, China) in light of standard protocol, to label CD4 and CD25 on cellular plasma membranes and Foxp3 in cellular nuclei. Subsequently, two peripheral blood lymphocyte subsets including CD4^+^ T cells and CD4^+^CD25^+^Foxp3^+^-positive Treg cells were defined on a flow cytometer (CytoFLEX, Beckman Coulter, USA), and data analysis was carried out with the CellQuest Pro software.

### 2.9 Enzyme-linked immunosorbent assay

Upon retrieval, rat lung tissues were homogenized with the lysis buffer on ice and then centrifuged, followed by collection of the supernatants for the following assessment of TNF-α and IL-1β with the ELISA kits, obtained from the Cloud-Clone Corporation (Wuhan, China), in accordance with the user’s manual.

### 2.10 Immunohistochemical analysis

In Brief, the lung tissues were harvested from sacrificed rats, fixed in 4% of paraformaldehyde solution, paraffin-embedded and sliced into 5 μm-thick sections. After dewaxing with xylene, anhydrous ethanol and gradient alcohol, the sections were treated with 3% hydrogen peroxide to remove endogenous peroxidase, followed by blockade with 3% bovine serum albumin (BSA) in TBST (TBS and 0.1% Tween 20) for 30 min at room temperature. After incubation with primary antibodies against TLR4 (1:100), phospho-NF-*κ*B p65 (1:100) and NLRP3 (1:50) respectively at 4°C overnight, the sections were treated with HRP-conjugated secondary antibody for 1 h, stained with chromogenic 3,3’-diaminobenzidine (DAB) solution and counterstained with hematoxylin, followed by dehydration, mounting and appraisal under a light microscope.

### 2.11 Cell culture

RAW264.7 mouse macrophage cell line, originally obtained from the American Type Culture Collection, was maintained by Dulbecco’s modified Eagle’s medium (DMEM), supplemented with 10% fetal bovine serum (FBS), 100 U/mL penicillin as well as 100 μg/mL streptomycin, at a humid atmosphere consisting of 5% CO_2_ and 95% air at 37°C.

### 2.12 MTT assay

RAW264.7 cells were seeded and grown in 96-well plates, and treated with QHSG at varying concentrations of 50, 75, 150, 200, 300, 400, 600 and 800 μg/mL for 24 h. Then, 200 μL of medium containing 10% of 3-(4,5-dimethylthiazol-2-yl)-2,5-diphenyl tetrazolium bromide (MTT) was added to each well, followed by incubation at 37°C for 4 h. Subsequently, 100 μL of dimethyl sulfoxide (DMSO) reagent was added to each well, followed by 15 min-shaking in the dark for dissolution of formazan crystals. Eventually, the absorbance was measured at 570 nm.

### 2.13 Stimulation of RAW264.7 cells with LPS

RAW264.7 cells were seeded and grown in 6-well plates, and treated by LPS at 1 μg/mL for 12 h in the presence or absence of QHSG at concentrations of 75 μg/mL, 150 μg/mL and 300 μg/mL or dexamethasone at 5 μg/mL, followed by further treatment with QHSG or dexamethasone for another 12 h. Then, the cells were harvested for qRT-PCR analysis and western blotting.

### 2.14 Real-time quantitative reverse transcription-polymerase chain reaction analysis

Total RNAs were extracted from rat lung tissues or mouse RAW264.7 cells utilizing the total RNA extraction kit (#R0032) (Beyotime, Shanghai, China), and then reversely transcribed to cDNAs with BeyoRT™ III cDNA first strand synthesis kit (#D7178M) (Beyotime, Shanghai, China). Later, qPCR was performed on BioRad CFX96™ instrument using 2 × Universal Blue SYBR Green qPCR Master Mix (#G3326-05) (Servicebio,Wuhan, China) according to the manufacturer’s instruction. β-actin was employed as an internal reference and the relative expressions of mRNAs were calculated by the 2^−ΔΔCt^ method. All primer sequences were listed in the **Tables 2** and **3**.

**Table 2:** The primers of qRT-PCR analysis for ALI Rats.

Genes	Primers	Sequences
Rat *β-actin*	Forward	5’-*GTGACGTTGACATCCGTAAAGAC*-3’
	Reverse	5’-*CAGGAGGAGCAATGATCTTGATC*-3’
Rat *IL-1β*	Forward	5’-*TCTTTGAAGAAGAGCCCGTCCTCTG*-3’
	Reverse	5’-*GGTCAGACAGCACGAGGCATTTTTG*-3’
Rat *IL-18*	Forward	5’-*CGAATCCCAGACCAGACTGATAAT*-3’
	Reverse	5’-*CAGGTGGATTCATTTCCTCAAAGG*-3’
Rat *IL-6*	Forward	5’-*TTTCTCTCCGCAAGAGACTTCCAG*-3’
	Reverse	5’-*TACTGGTCTGTTGTGGGTGGTATC*-3’
Rat *TNF-α*	Forward	5’-*GGAAAGCATGATCCGAGATGTGGA*-3’
	Reverse	5’-*CCCATTTGGGAACTTCTCCTCCTTG*-3’
Rat *IFN-γ*	Forward	5’-*AACTGGCAAAAGGACGGTAACACG*-3’
	Reverse	5’-*GTTGTTGCTGATGGCCTGGTTGTC*-3’
Rat *TLR4*	Forward	5’-*CCCTGCATAGAGGTACTTCCTA*-3’
	Reverse	5’-*CAGCCACTGAAGTTGTGAGAA*-3’
Rat *NLRP3*	Forward	5’-*GTTCTTCCAGACTGGTGAACTGC*-3’
	Reverse	5’-*TCCCCTAGAGTATTGTCACTGAGG*-3’
Rat *ASC*	Forward	5’-*CCCCATAGACCTCACTGATAAAC*-3’
	Reverse	5’-*CAGCTCCAGACTCTTCCATAATC*-3’
Rat *GSDMD*	Forward	5’-*CTGAGTCTCAAGTCAGATGGAACCA*-3’
	Reverse	5’-*TGGTCCTGTAAAATCCTCCCGATG*-3’
Rat *CASP1*	Forward	5’-*TGCCCTCATTATCTGCAACACAG*-3’
	Reverse	5’-*CTTTTGTCATCTCCAGAGCTGTGAG*-3’

**Table 3:** The primers of qRT-PCR analysis for mouse RAW264.7 cells.

Genes	Primers	Sequences
Mouse *β-actin*	Forward	5’-*TAGGCACCAGGGTGTGATG*-3’
	Reverse	5’-*GTGGTGCCAGATCTTCTCCA*-3’
Mouse *IL-1β*	Forward	5’-*CTTTGAAGAAGAGCCCATCCTCTGTG*-3’
	Reverse	5’-*GGTGGAGAGCTTTCAGCTCATATGG*-3’
Mouse *IL-18*	Forward	5’-*CTGAAGAAAATGGAGACCTGGA*-3’
	Reverse	5’-*CAGTCATATCCTCGAACACAGG*-3’
Mouse *IL-6*	Forward	5’-*TCCTCTCTGCAAGAGACTTCCATC*-3’
	Reverse	5’-*GTGGTATAGACAGGTCTGTTGGGA*-3’
Mouse *TNF-α*	Forward	5’-*GCCTATGTCTCAGCCTCTTCTCA*-3’
	Reverse	5’-*TTGGGAACTTCTCATCCCTTTGG*-3’
Mouse *IFN-γ*	Forward	5’-*GGAACTGGCAAAAGGATGGTGACATG*-3’
	Reverse	5’-*CGCTTATGTTGTTGCTGATGGCCTG*-3’
Mouse *TLR4*	Forward	5’-*GGCTGGATTTATCCAGGTGTGA*-3’
	Reverse	5’-*TGTTAGTCCAGAGAAACTTCCTGG*-3’
Mouse *NLRP3*	Forward	5’-*AGGCAGATCACTTGGATCTAGC*-3’
	Reverse	5’-*TACACGTGTCATTCCACTCTGG*-3’
Mouse *ASC*	Forward	5’-*TACAGCCAGAACAGGACACTTTG*-3’
	Reverse	5’-*GCTGAAGAGCTTCCTCATCTTGTC*-3’
Mouse *GSDMD*	Forward	5’-*CCTTCAGCAGCCTGAGAACAA*-3’
	Reverse	5’-*CTTGCCTTCACCCTTCAAGCA*-3’
Mouse *CASP1*	Forward	5’-*CACAGCTCTGGAGATGGTGAAA*-3’
	Reverse	5’-*GCTTGGGCACTTCAAAGTGTTC*-3’

### 2.15 Western blotting

Total proteins were extracted from rat lung tissues or mouse RAW264.7 cells on ice with radioimmunoprecipitation assay buffer, containing protease inhibitors and phosphatase inhibitors, while the nuclear proteins were extracted with nuclear protein extraction kit (#R0050-50T) (Solarbio, Beijing, China) according to the manufacturer’s instruction, followed by quantification using BCA protein assay kit (#P0012) (Beyotime, Shanghai, China). Subsequently, the proteins were fractionated by sodium dodecyl sulfate-polyacrylamide gel electrophoresis and transferred to polyvinylidene difluoride membranes. After being blocked with TBST containing 3% BSA at room temperature for 45 min, the membranes were then incubated with primary antibodies at 1:1000 dilution at 4 °C overnight. After being washed with TBST thrice, the membranes were incubated with HRP-conjugated secondary antibodies at 1:2000 dilution for 1.5 h at room temperature. Finally, the immunoreactive bands were visualized with enhanced chemiluminescence kit on ChemiDoc XRS+ system (Bio-Rad, California, USA), and analyzed with the Image Lab software. The protein abundances were expressed as the relative levels versus the controls (23–25).

### 2.16 Statistical analysis

All experiments were repeated at least 3 times. The data were analyzed by one-way analysis of variance (ANOVA) with Tukey’s *post hoc* test, using the SPSS and GraphPad Prism software. The results were presented as mean ± SEM (standard error of the mean) unless otherwise indicated, with the statistical significance set at a two-sided value of P < 0.05.

## 3 Results

### 3.1 Analysis of the chemical composition of QHSG by HPLC

**Figure 1:**
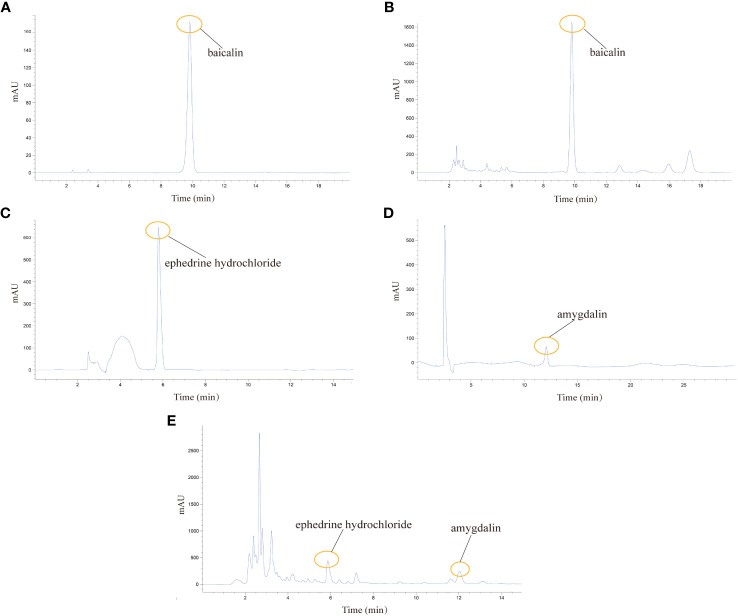
Analysis of the chemical composition of QHSG by HPLC. **(A)** The chromatogram of baicalin standard. **(B)** Identification of baicalin in QHSG. **(C)** The chromatogram of ephedrine hydrochloride standard. **(D)** The chromatogram of amygdalin standard. **(E)** Identification of ephedrine hydrochloride and amygdalin in QHSG.

Being crucial constituents of QHSG, baicalin, ephedrine hydrochloride and amygdalin may serve as the chemical makers of QHSG. With baicalin, ephedrine hydrochloride and amygdalin as standard substances, these three compounds were identified in QHSG by HPLC under two distinct analytic conditions (**Figure 1**). The retention times of baicalin, ephedrine hydrochloride and amygdalin were 9.814 min, 5.864 min and 12.037 min, respectively. These data ensured the quality of used QHSG was standard and the results of the following study on QHSG were repeatable.

### 3.2 QHSG alleviates the pathological features of LPS-induced acute lung injury in rats

**Figure 2:**
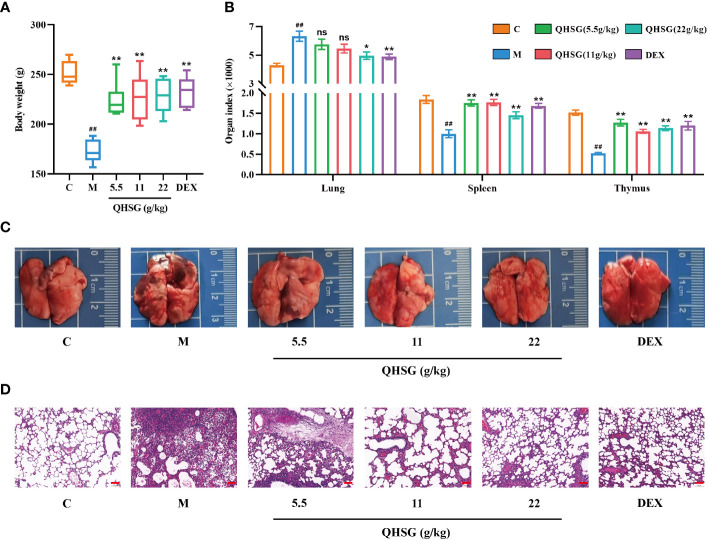
QHSG alleviates the pathological features of LPS-induced acute lung injury in rats. **(A)** The body weights of the rats with LPS-induced ALI, treated by QHSG at 5.5 g/kg, 11 g/kg and 22 g/kg or dexamethasone at 2 mg/kg. N=6. **(B)** The indices of organs, including lung, spleen and thymus, of the rats with LPS-induced ALI, treated by QHSG at 5.5 g/kg, 11 g/kg and 22 g/kg or dexamethasone at 2 mg/kg. N=6. **(C)** Representative photographs of the lungs of the rats with LPS-induced ALI, treated by QHSG at 5.5 g/kg, 11 g/kg and 22 g/kg or dexamethasone at 2 mg/kg. **(D)** H&E staining analysis of the lung tissues of the rats with LPS-induced ALI, treated by QHSG at 5.5 g/kg, 11 g/kg and 22 g/kg or dexamethasone at 2 mg/kg. Scale bars = 100 μm. N=3. C, the normal control; M, the model rats; ns, not significant. ^##^ *p*< 0.01 versus the normal control. *p < 0.05 and **p < 0.01 versus the model rats.

Firstly, the model of LPS-induced acute lung injury was established in rats, followed by treatment with QHSG at 5.5 g/kg, 11 g/kg and 22 g/kg or dexamethasone at 2 mg/kg. It was found that the growths of the rats were sharply restrained by LPS, which were negated by treatment with QHSG and dexamethasone, resulting in significant body weight gain in the rats (**Figure 2A**). Meanwhile, LPS was shown to augment the lung index but reduce the indices of spleen and thymus of the rats, suggestive of pulmonary edema and disorders of the immune system in ALI rats, which were ameliorated by treatment with QHSG and dexamethasone (**Figure 2B**). These results were partially corroborated by anatomical examination of the lungs, unveiling that QHSG and dexamethasone relieved pulmonary edema and inflammatory exudation of the rats with LPS-induced acute lung injury (**Figure 2C**). Also, H&E staining analysis of the lung tissues demonstrated that QHSG and dexamethasone substantially suppressed the pathological responses of the ALI rats including pulmonary edema, inflammatory infiltration and necrosis. To sum up, QHSG may alleviate the pathological features of LPS-induced acute lung injury in rats.

### 3.3 QHSG reverses the alteration in white blood cell profile of the rats with LPS-induced acute lung injury

**Figure 3:**
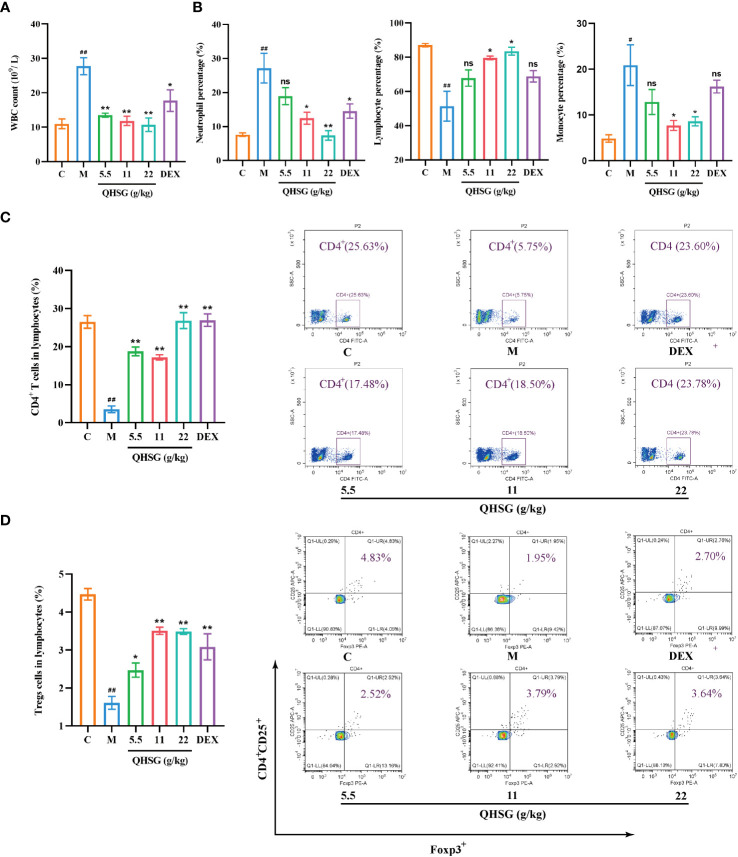
QHSG reverses the alteration in white blood cell subset panel of the rats with LPS-induced acute lung injury. **(A)** WBC count of the rats with LPS-induced ALI, treated by QHSG at 5.5 g/kg, 11 g/kg and 22 g/kg or dexamethasone at 2 mg/kg. N=4. **(B)** The percentages of peripheral blood neutrophils, lymphocytes and monocytes in WBC of the rats with LPS-induced ALI, treated by QHSG at 5.5 g/kg, 11 g/kg and 22 g/kg or dexamethasone at 2 mg/kg. N=4. **(C)** The percentage of CD4^+^ T cells in the lymphocytes of the rats with LPS-induced ALI, treated by QHSG at 5.5 g/kg, 11 g/kg and 22 g/kg or dexamethasone at 2 mg/kg. N=4. **(D)** The percentage of CD4^+^CD25^+^Foxp3^+^-positive Treg cells in the lymphocytes of the rats with LPS-induced ALI, treated by QHSG at 5.5 g/kg, 11 g/kg and 22 g/kg or dexamethasone at 2 mg/kg. N=4. C, the normal control; M, the model rats; ns, not significant. ^#^ *p*< 0.05 and ^##^ *p*< 0.01 versus the normal control. *p < 0.05 and **p < 0.01 versus the model rats.

Next, the influence of QHSG on the peripheral white blood cell subset panel of ALI rats was investigated. It was discovered that QHSG at 5.5 g/kg, 11 g/kg and 22 g/kg all drastically weakened LPS-caused increase in WBC count of the rats (**Figure 3A**). In comparison with the normal control, the percentages of peripheral blood neutrophils and monocytes in WBC were elevated, whilst lymphocyte percentage in WBC was lowered, in the rats with LPS-induced acute lung injury, which were negated by medication with QHSG and dexamethasone (**Figure 3B**), suggesting that QHSG inhibited the inflammatory response in ALI rats.

Treg cells are a subset of CD4^+^ T lymphocytes that express the IL-2Rα (CD25) and suppress autoimmune disease. Transcription factor forkhead box P3 (Foxp3) controls gene expression of Treg cells. LPS was shown to reduce the percentages of CD4^+^ T cells and CD4^+^CD25^+^Foxp3^+^-positive Treg cells in the lymphocytes of the rats, which were significantly reversed by treatment with QHSG at 5.5 g/kg, 11 g/kg and 22 g/kg and dexamethasone at 2 mg/kg (**Figures 3C, D**), manifesting that QHSG combatted LPS-induced ALI by regulation of the immune function in the rats.

### 3.4 QHSG impedes the production of inflammatory cytokines in LPS-induced acute lung injury of the rats

**Figure 4:**
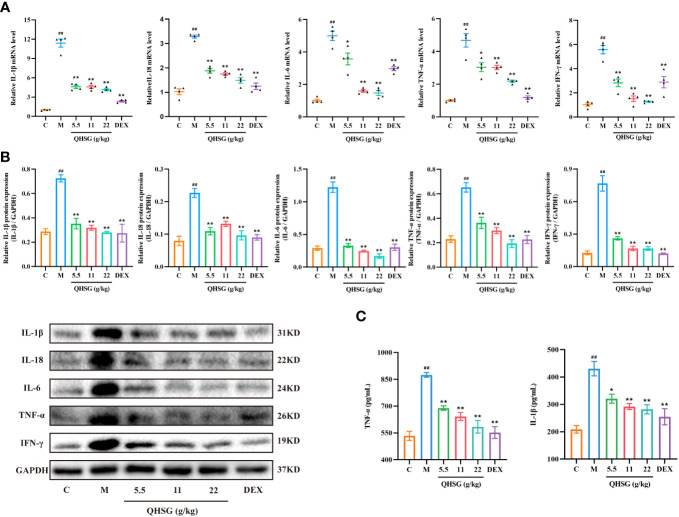
QHSG impedes the production of inflammatory cytokines in LPS-induced acute lung injury of the rats. **(A)** The qRT-PCR analyses of the mRNA levels of *IL-1β*, *IL-18*, *IL-6*, *TNF-α* and *IFN-γ* in the lung tissues of the rats with LPS-induced ALI, treated by QHSG at 5.5 g/kg, 11 g/kg and 22 g/kg or dexamethasone at 2 mg/kg. N=4. **(B)** Western blotting of the protein expressions of IL-1β, IL-18, IL-6, TNF-α and IFN-γ in the lung tissues of the rats with LPS-induced ALI, treated by QHSG at 5.5 g/kg, 11 g/kg and 22 g/kg or dexamethasone at 2 mg/kg. N=3. **(C)** ELISA of TNF-α and IL-1β levels in the lung tissues of the rats with LPS-induced ALI, treated by QHSG at 5.5 g/kg, 11 g/kg and 22 g/kg or dexamethasone at 2 mg/kg. N=4. C, the normal control; M, the model rats. ^##^ *p*< 0.01 versus the normal control. *p < 0.05 and **p < 0.01 versus the model rats.

Subsequently, the effect of QHSG on the pro-inflammatory mediators in LPS-induced acute lung injury of the rats was appraised. The qRT-PCR analyses presented that QHSG drastically diminished the mRNA levels of inflammatory cytokines, including *IL-1β*, *IL-18*, *IL-6*, *TNF-α* and *IFN-γ*, in the lung tissues of the rats with LPS-induced ALI, in comparison with the normal control (**Figure 4A**), which were consistent with the results of western blot analyses, manifesting that IL-1β, IL-18, IL-6, TNF-α and IFN-γ protein expressions were immensely hampered by QHSG in the lung tissue of LPS-induced ALI rats (**Figure 4B**). Also, ELISA demonstrated that the augmentation of TNF-α and IL-1β levels in the lung tissues of LPS-induced ALI rats, relative to the normal control, was depressed by QHSG in a dose-dependent manner (**Figure 4C**). Collectively, it was substantiated that QHSG repressed LPS-induced acute lung injury of the rats, by mitigation of the inflammatory cytokine levels.

### 3.5 QHSG down-regulates TLR4/NF-κB signaling cascade in LPS-induced acute lung injury of the rats

**Figure 5:**
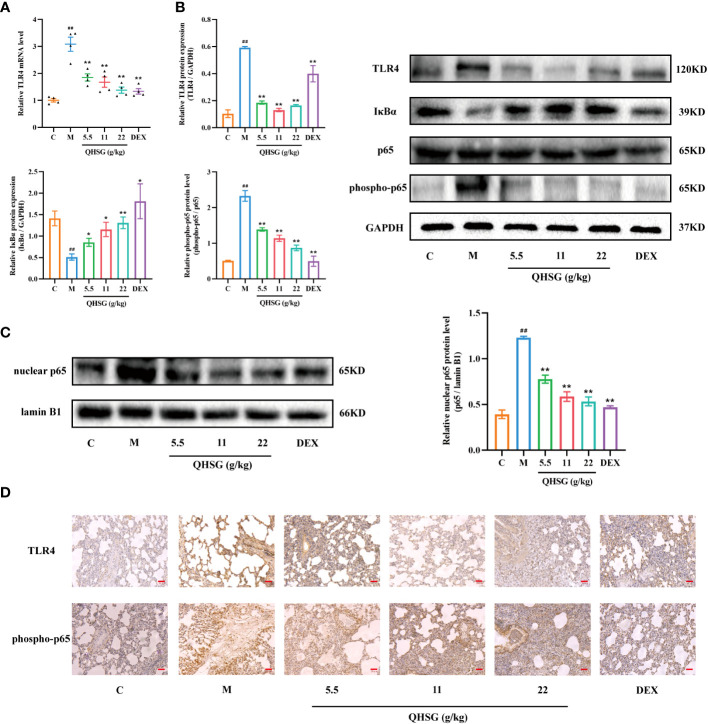
QHSG down-regulates TLR4/NF-*κ*B signaling cascade in LPS-induced acute lung injury of the rats. **(A)** The qRT-PCR analysis of *TLR4* mRNA level in the lung tissues of the rats with LPS-induced ALI, treated by QHSG at 5.5 g/kg, 11 g/kg and 22 g/kg or dexamethasone at 2 mg/kg. N=4. **(B)** Western blotting of the protein expressions of TLR4, I*κ*Bα, p65 and phospho-p65 in the lung tissues of the rats with LPS-induced ALI, treated by QHSG at 5.5 g/kg, 11 g/kg and 22 g/kg or dexamethasone at 2 mg/kg. N=3. **(C)** Western blotting of nuclear p65 protein level in the lung tissues of the rats with LPS-induced ALI, treated by QHSG at 5.5 g/kg, 11 g/kg and 22 g/kg or dexamethasone at 2 mg/kg. N=3. **(D)** IHC analyses of protein levels of TLR4 and phospho-p65 in the lung tissues of the rats with LPS-induced ALI, treated by QHSG at 5.5 g/kg, 11 g/kg and 22 g/kg or dexamethasone at 2 mg/kg. Scale bars = 20 μm. N=3. C, the normal control; M, the model rats. ^##^ *p*< 0.01 versus the normal control. *p < 0.05 and **p < 0.01 versus the model rats.

TLR4, a transmembrane protein, belongs to the pattern recognition receptor (PRR) family. Upon activation by LPS (26), TLR4 initiates an intracellular NF-*κ*B signaling pathway, resulting in the production of inflammatory cytokines, which is crucial for the activation of the innate immune system (27). In comparison with the normal control, LPS strengthened TLR4 mRNA transcription and protein translation in the lung tissues of ALI rats (**Figures 5A, B**), which was abrogated by treatment with QHSG (**Figures 5A, B**). Also, the protein expression of I*κ*Bα, an inhibitor of NF-*κ*B activation, was dose-dependently intensified in QHSG-treated rats with LPS-induced ALI, manifesting that QHSG may restrain NF-*κ*B activation in LPS-induced acute lung injury (**Figure 5B**). It is well documented that phosphorylation of p65 promotes NF-*κ*B activation (28). QHSG was discovered to attenuate the phospho-p65 protein level of the lung tissues of ALI rats in a dose-dependent mode (**Figure 5B**). Without influence on total p65 protein expression in the lung tissues of ALI rats, QHSG reduced the nuclear p65 protein level in a dose-dependent pattern, suggesting that QHSG impeded the nuclear translocation of p65 to suppress downstream target gene expression in LPS-induced acute lung injury (**Figures 5B, C**). IHC analyses revealed that QHSG lowered TLR4 and phospho-p65 protein levels in the lung tissues of ALI rats (**Figure 5D**). On the whole, QHSG may alleviate LPS-incurred acute lung injury of the rats by down-regulation of TLR4/NF-*κ*B signaling pathway activity.

### 3.6 QHSG inhibits NLRP3 inflammasome activation in LPS-induced acute lung injury of the rats

NLRP3 inflammasome is present in immune cells of the innate immune system including macrophages and also in epithelial cells. Activation of NLRP3 inflammasome is highly implicated in the pathogenesis of ALI. In addition, IL-1β and IL-18 released following inflammasome activation can induce IFN-γ secretion and natural killer cell activation (29). Also, TLRs and NLRs may upregulate the expression of inflammatory cytokines such as TNF, IL-6 and IL-12 through NF-*κ*B signaling.

**Figure 6:**
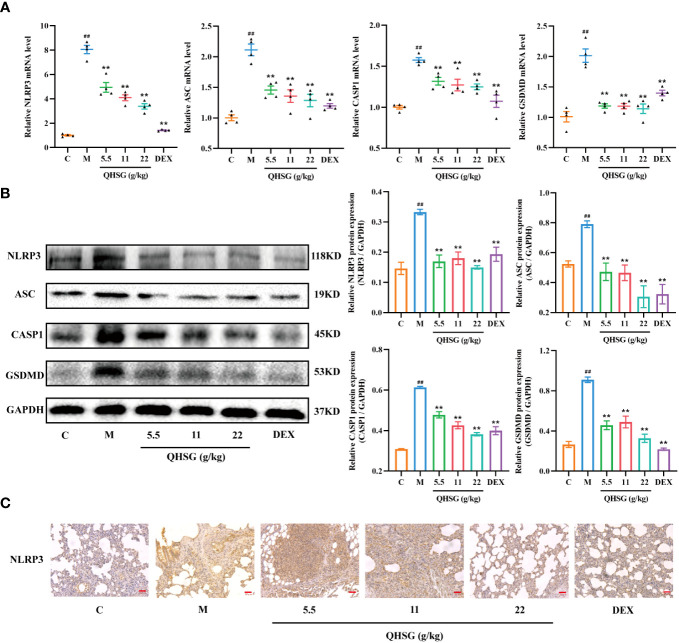
QHSG inhibits NLRP3 inflammasome activation in LPS-induced acute lung injury of the rats. **(A)** The qRT-PCR analyses of the mRNA levels of *NLRP3*, *ASC*, *CASP1* and *GSDMD* in the lung tissues of the rats with LPS-induced ALI, treated by QHSG at 5.5 g/kg, 11 g/kg and 22 g/kg or dexamethasone at 2 mg/kg. N=4. **(B)** Western blotting of the protein expressions of NLRP3, ASC, CASP1 and GSDMD in the lung tissues of the rats with LPS-induced ALI, treated by QHSG at 5.5 g/kg, 11 g/kg and 22 g/kg or dexamethasone at 2 mg/kg. N=3. **(C)** IHC analysis of NLRP3 protein level in the lung tissues of the rats with LPS-induced ALI, treated by QHSG at 5.5 g/kg, 11 g/kg and 22 g/kg or dexamethasone at 2 mg/kg. Scale bars = 20 μm. N=3. C, the normal control; M, the model rats. ^##^ *p*< 0.01 versus the normal control. *p < 0.05 and **p < 0.01 versus the model rats.

In the present study, LPS enhanced the mRNA and protein expressions of NLRP3, ASC, CASP1 and GSDMD in the lung tissues of ALI rats, compared with the normal control (**Figures 6A, B**). The qRT-PCR and western blot analyses presented that treatment of LPS-induced ALI rats with QHSG at dosages of 5.5 g/kg, 11 g/kg and 22 g/kg caused significant slump in the mRNA transcriptions and protein translations of NLRP3, ASC, CASP1 and GSDMD in the lung tissues (**Figures 6A, B**), which was reinforced by IHC assay, showing NLRP3 protein level was markedly lowered in the lung tissues of QHSG-treated ALI rats than that in the model rats (**Figure 6C**). All in all, QHSG may relieve LPS-elicited acute lung injury of the rats by inhibition of NLRP3 inflammasome activation, which is a critical process to pro-inflammatory pyroptosis.

### 3.7 QHSG hinders the generation of inflammatory cytokines in LPS-stimulated mouse macrophages

**Figure 7:**
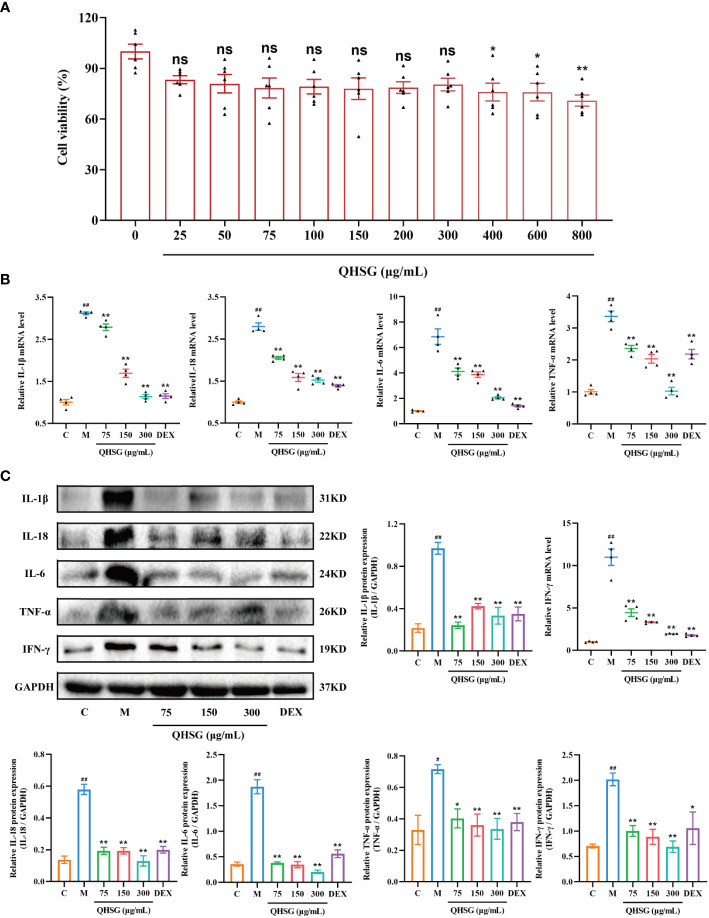
QHSG hinders the generation of inflammatory cytokines in LPS-stimulated mouse macrophages. **(A)** MTT assay of the viability of RAW264.7 cells treated with QHSG at concentrations ranging from 25 μg/mL to 800 μg/mL for 24 **(h)** N=6. **(B)** The qRT-PCR analyses of the mRNA levels of *IL-1β*, *IL-18*, *IL-6*, *TNF-α* and *IFN-γ* in LPS-stimulated RAW264.7 cells treated with QHSG at 75 μg/mL, 150 μg/mL and 300 μg/mL or DEX at 5 μg/mL. N=4. **(C)** Western blotting of the protein expressions of IL-1β, IL-18, IL-6, TNF-α and IFN-γ in LPS-stimulated RAW264.7 cells treated with QHSG at 75 μg/mL, 150 μg/mL and 300 μg/mL or DEX at 5 μg/mL. N=3. C, the normal control; M, LPS-stimulated RAW264.7 cells; ns, not significant. ^#^ *p*< 0.05 and ^##^ *p*< 0.01 versus the normal control. *p < 0.05 and **p < 0.01 versus LPS-stimulated RAW264.7 cells.

In order to verify the efficacy of QHSG on LPS-induced acute lung injury in the rats, the potency of QHSG on RAW264.7 mouse macrophages challenged by LPS was investigated. Firstly, QHSG was unveiled to exert no toxicity to RAW264.7 mouse macrophages without LPS stimulation, as QHSG did not influence the viability of RAW264.7 cells at concentrations of up to 800 μg/mL (**Figure 7A**). Therefore, the concentrations of 75 μg/mL, 150 μg/mL and 300 μg/mL were employed for the following study on the effect of QHSG on LPS-stimulated macrophages *in vitro*.

Compared with the normal control, RAW264.7 cells incited by LPS were shown to produce more inflammatory cytokines including IL-1β, IL-18, IL-6, TNF-α and IFN-γ, as the mRNA and protein levels of these cytokines were higher in LPS-treated RAW264.7 cells than those in unstimulated cells (**Figures 7B, C**). The simulating effect of LPS was antagonized by QHSG, as treatment with QHSG at 75 μg/mL, 150 μg/mL and 300 μg/mL substantially deterred the mRNA transcription and protein translation of IL-1β, IL-18, IL-6, TNF-α and IFN-γ in LPS-challenged RAW264.7 cells (**Figures 7B, C**), suggesting that QHSG may weaken inflammatory response in LPS-stimulated mouse macrophages by encumbering the generation of inflammatory cytokines in macrophages.

### 3.8 QHSG lessens TLR4/NF-κB signaling cascade in inflammatory response of LPS-stimulated mouse macrophages

**Figure 8:**
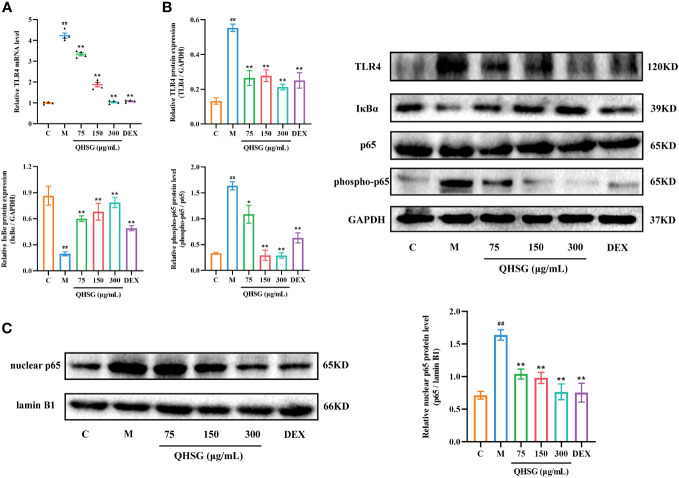
QHSG lessens TLR4/NF-*κ*B signaling cascade in inflammatory response of LPS-stimulated mouse macrophages. **(A)** The qRT-PCR analysis of *TLR4* mRNA level in LPS-stimulated RAW264.7 cells treated with QHSG at 75 μg/mL, 150 μg/mL and 300 μg/mL or DEX at 5 μg/mL. N=4. **(B)** Western blotting of the protein expressions of TLR4, I*κ*Bα, p65 and phospho-p65 in LPS-stimulated RAW264.7 cells treated with QHSG at 75 μg/mL, 150 μg/mL and 300 μg/mL or DEX at 5 μg/mL. N=3. **(C)** Western blotting of nuclear p65 protein level in LPS-stimulated RAW264.7 cells treated with QHSG at 75 μg/mL, 150 μg/mL and 300 μg/mL or DEX at 5 μg/mL. N=3. C, the normal control; M, LPS-stimulated RAW264.7 cells. ^##^ *p*< 0.01 versus the normal control. *p < 0.05 and **p < 0.01 versus LPS-stimulated RAW264.7 cells.

To unravel the mechanism by which QHSG hinders the generation of inflammatory cytokines in LPS-stimulated mouse macrophages, the effectiveness of QHSG on TLR4/NF-*κ*B signaling pathway activity in LPS-challenged RAW264.7 cells was determined. Compared with the normal control, LPS gave rise to the increase in TLR4 mRNA transcription and protein translation in LPS-challenged RAW264.7 cells (**Figures 8A, B**), which was eradicated by treatment with QHSG at 75 μg/mL, 150 μg/mL and 300 μg/mL (**Figures 8A, B**). Also, the protein expression of NF-*κ*B inhibitor I*κ*Bα was concentration-dependently strengthened by treatment with QHSG in LPS-challenged RAW264.7 cells, hinting that QHSG may repress aberrant activation of NF-*κ*B in LPS-incited mouse macrophages (**Figure 8B**).

It is well known that phosphorylation of p65 potentiates NF-*κ*B activation. QHSG was unveiled to strikingly lower the phospho-p65 protein level in LPS-spurred RAW264.7 cells (**Figure 8B**). Without influence on total p65 protein expression in LPS-challenged RAW264.7 cells, QHSG lessened the nuclear p65 protein level in a concentration-dependent mode, manifesting that QHSG dampened the nuclear translocation of p65 to encumber downstream gene expression in LPS-provoked macrophages (**Figures 8B, C**). Overall, QHSG may relieve inflammatory response of LPS-stimulated mouse macrophages by down-regulation of TLR4/NF-*κ*B signaling pathway activity.

### 3.9 QHSG weakens NLRP3 inflammasome activation in inflammatory response of LPS-stimulated mouse macrophages

**Figure 9:**
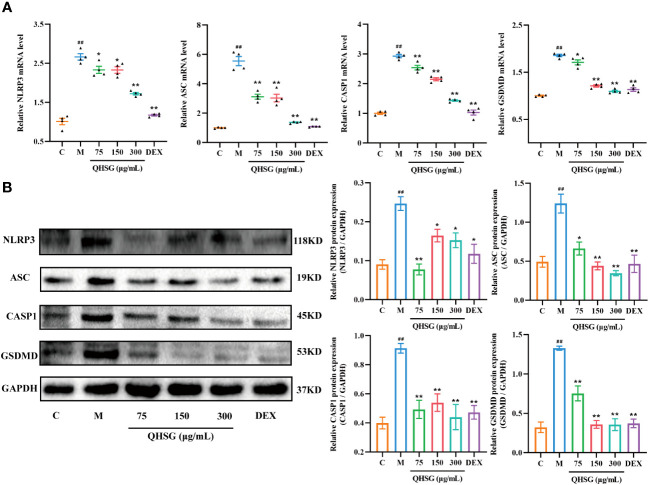
QHSG weakens NLRP3 inflammasome activation in inflammatory response of LPS-stimulated mouse macrophages. **(A)** The qRT-PCR analyses of the mRNA levels of *NLRP3*, *ASC*, *CASP1* and *GSDMD* in LPS-stimulated RAW264.7 cells treated with QHSG at 75 μg/mL, 150 μg/mL and 300 μg/mL or DEX at 5 μg/mL. N=4. **(B)** Western blotting of the protein expressions of NLRP3, ASC, CASP1 and GSDMD in LPS-stimulated RAW264.7 cells treated with QHSG at 75 μg/mL, 150 μg/mL and 300 μg/mL or DEX at 5 μg/mL. N=3. C, the normal control; M, LPS-stimulated RAW264.7 cells. ^##^ *p*< 0.01 versus the normal control. *p < 0.05 and **p < 0.01 versus LPS-stimulated RAW264.7 cells.

It is well documented that NLRP3 inflammasome is highly present in immune cells of the innate immune system including macrophages. In comparison with the normal control, the mRNA and protein expressions of NLRP3, ASC, CASP1 and GSDMD were augmented in LPS-challenged RAW264.7 cells (**Figures 9A, B**), which were eliminated by QHSG, as the qRT-PCR and western blot analyses demonstrated that treatment of LPS-stimulated RAW264.7 cells with QHSG at concentrations of 75 μg/mL, 150 μg/mL and 300 μg/mL resulted in significant decline in the mRNA transcriptions and protein translations of NLRP3, ASC, CASP1 and GSDMD in mouse macrophages (**Figures 9A, B**), consistent with the effectiveness of QHSG on ALI rats. All in all, QHSG may alleviate the inflammatory response of LPS-stimulated mouse macrophages by repressing the abnormal activation of NLRP3 inflammasome.

## 4 Discussion

Acute lung injury is an extremely serious inflammatory disease. Despite unremitting efforts, effective and safe therapy for ALI is still scarce clinically. Therefore, it is necessary to innovate novel drugs to tackle this kind of affliction. In recent years, there has been a growing interest in the exploitation of Chinese materia medica for medication of ALI. Traditional Chinese medicine prescriptions are known for their multicomponent and multitarget nature, which facilitate the interaction between each component to effectively enhance efficacy and reduce toxicity (30). Several Chinese herbal formulas, such as Xuanfei Baidu formula, Ge-Gen-Qin-Lian decoction, Danhong injection, and Re-Du-Ning injection, have been reported to significantly alleviate the symptoms of ALI (31–34).

**Figure 10:**
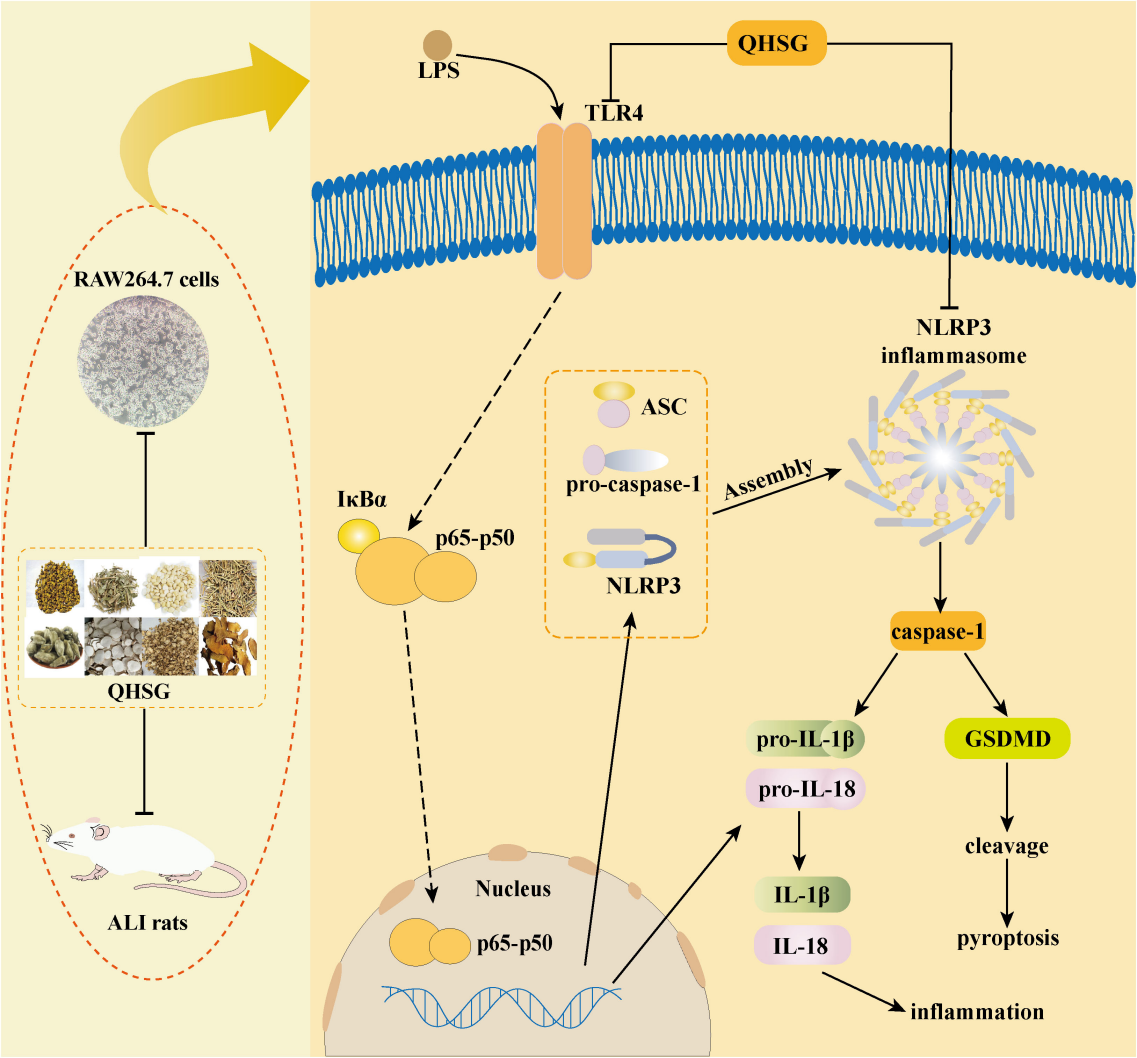
Schematic representation showing QHSG may resolve acute lung injury through its anti-inflammation and immunoregulation, by down-regulating TLR4/NF-*κ*B signaling cascade and inhibiting NLRP3 inflammasome activation. QHSG ameliorated ALI by alleviating the pathological features, reversing the alteration in white blood cell profile and impeding the production of inflammatory cytokines in LPS-induced ALI of the rats, through down-regulation of TLR4/NF-*κ*B signaling cascade and inhibition of NLRP3 inflammasome activation. Also, QHSG hindered the generation of inflammatory cytokines in LPS-stimulated RAW264.7 mouse macrophages, by lessening TLR4/NF-*κ*B signaling pathway activity and weakening NLRP3 inflammasome activation. *(Drawn with Adobe Illustrator 2021 software)*.

In the present study, with the rat model of LPS-induced ALI, QHSG was unveiled to ameliorate ALI by alleviating the pathological features, reversing the alteration in white blood cell profile and impeding the production of inflammatory cytokines in LPS-induced ALI of the rats through down-regulation of TLR4/NF-*κ*B signaling cascade and inhibition of NLRP3 inflammasome activation. In LPS-stimulated RAW264.7 mouse macrophages, QHSG was discovered to hinder the generation of inflammatory cytokines by lessening TLR4/NF-*κ*B signaling pathway activity and weakening NLRP3 inflammasome activation (**Figure 10**).

Baicalin, amygdalin and ephedrine are the main components of *Scutellariae radix*, *Armeniacae semen amarum* and *Ephedrae herba*, respectively. Previous studies have found that they have dramatically inhibitory effects on excessive inflammatory response (35–37). Hence, we carried out HPLC analysis to determine the quality of QHSG with amygdalin, baicalin and ephedrine as standard substances and found that amygdalin, baicalin and ephedrine were present in QHSG. Thus, we raised the hypothesis that QHSG may have certain potency for treating ALI.

White blood cells, neutrophils, lymphocytes, and monocytes are important indicators of inflammation and immune function (38). In this study, WBC count, the percentages of peripheral blood neutrophils and monocytes in WBC were increased, while lymphocyte percentage in WBC was decreased, in the rats with LPS-induced ALI, indicating that the rat lungs suffered acute infection or tissue necrosis and other pathological damage, leading to inflammatory reaction and immune response. Intriguingly, QHSG treatment significantly alleviated these symptoms. Alveolar inflammatory infiltration, pulmonary edema and other lung tissue lesions are the main pathological characteristics of ALI (39). In LPS-induced ALI model, changes in lung index reflected pulmonary edema, while lung photographs and H&E staining directly manifested lung injury. Pharmacological studies showed that QHSG significantly reduced pulmonary edema and alleviated inflammatory infiltration, revealing that QHSG may have a therapeutic effect on ALI.

Massive release of inflammatory cytokines and immune dysfunction are the immanent causes of the occurrence and development of ALI (40). Existing evidence reveals that macrophages can phagocytose pathogens and damaged tissue, while secreting a large number of inflammatory cytokines and chemotactic cytokines, which play an important role in inflammatory disease (41). RAW264.7 mouse macrophage cell line has been widely used as an inflammation model *in vitro* to explore the anti-inflammatory effects of drugs (42). In this study, QHSG significantly decreased IL-1β, IL-18, IL-6, TNF-α and IFN-γ mRNA levels and protein expressions in RAW264.7 cells, suggesting that QHSG could inhibit the release of inflammatory cytokines, which was also consistent with animal experiments *in vivo*. In addition, TNF-α and IL-1β levels in the lung tissues of the rats with LPS-caused ALI were dramatically lowered after QHSG treatment. Overall, QHSG may play a crucial suppressive role in inflammatory response.

T lymphocytes are a crucial subset of lymphocytes that play a significant role in regulating immunity to maintain homeostasis (43). In the present study, the administration of LPS resulted in a decrease in body weight in rats, indicating that LPS inhibited the growth and development of the rats, which was counteracted by treatment with QHSG. Additionally, the spleen index and thymus index were increased after QHSG treatment, suggesting that QHSG may have a supportive impact on immune function. Further investigation revealed that LPS administration significantly reduced the proportion of CD4^+^ T cells and CD4^+^CD25^+^Foxp3^+^-positive Treg cells, leading to impaired immune function. However, this effect was reversed by QHSG treatment, resulting in the restoration of immune function to normal levels. CD4^+^ T cells and CD4^+^CD25^+^Foxp3^+^-positive Treg cells are well-known subsets of helper T cells and regulatory T cells, respectively. Helper T cells secrete cytokines to regulate and assist immune responses, while regulatory T cells play a crucial role in maintaining immune homeostasis and immune tolerance (44, 45). Importantly, QHSG treatment increased the proportion of helper T cells and regulatory T cells in the body, thereby strengthening immunity and enabling the body to cope with the severe injury and immunological disturbance caused by LPS infection.

It is well established that the TLR4/NFkB pathway plays a crucial role in the development of acute lung injury (46). TLR4, a member of the Toll-like receptor family, is primarily found on the surface of immune effector cells, epithelial cells and endothelial cells. TLR4 activates innate immunity in a ligand-dependent manner (47). LPS binds to TLR4 with the assistance of MD2 and CD14, leading to the activation of NF-*κ*B signaling pathway through the MyD88 intermediate (48). NF-*κ*B is a transcription factor that plays a vital role in inflammatory and immune responses, by potentiating the production of various inflammatory mediators and cytokines (49). In the classical NF-*κ*B signaling pathway, stimulation and activation of I*κ*B kinase (IKK) result in the degradation-causing phosphorylation of I*κ*Bα in the cytoplasm, which leads to the release and reactivation of NF-*κ*B heterodimer (p65-p50). Subsequently, the heterodimer undergoes post-translational modification and is transported to the nucleus, where p65-p50 interacts with DNA to initiate the transcription of target genes and release of inflammatory cytokines, ultimately causing inflammatory response (50). In both *in vivo* and *in vitro* studies, QHSG was found to suppress the mRNA level and protein expression of TLR4, indicating a potential inhibition of TLR4 signaling cascade. Additionally, QHSG up-regulated the protein expression of I*κ*Bα and down-regulated the levels of phospho-NF-*κ*B p65 and nuclear NF-*κ*B p65. These results suggest that QHSG can inhibit the phosphorylation of I*κ*Bα and NF-*κ*B p65, as well as the nuclear translocation of NF-*κ*B p65. Our results are in agreement with other reports that baicalin, a bioactive component of QHSG, inhibits the secretion of inflammatory factors in LPS-induced acute lung injury in rats by suppressing the TLR4/myeloid differentiation factor 88 (MyD88)/NF-*κ*B signaling pathway (51). Also, baicalin relieves *mycoplasma pneumoniae* infection−induced lung injury in mice by regulating microRNA−221 to inhibit the TLR4/NF-*κ*B signaling pathway (52). To sum up, QHSG revealed the ability to prevent LPS-induced ALI by inhibiting the activity of the TLR4/NF-*κ*B signaling pathway.

There is increasing evidence to suggest that the abnormal activation of NLRP3 inflammasome is closely associated with the deterioration of ALI (53). NLRP3 inflammasome is implicated in the function of the innate immune system (54). Activation of the TLR4/NF-*κ*B signaling pathway results in the upregulation of NLRP3, pro-IL-1β and pro-IL-18 (55). Additionally, various stimuli such as PAMP/damage-associated molecular pattern (DAMP) indirectly activate NLRP3, leading to the assembly of NLRP3 inflammasome and activation of CASP1, which triggers the synthesis and secretion of IL-1β and IL-18 (56). Simultaneously, activated CASP1 cleaves the N-terminal sequence of GSDMD, giving rise to the production of GSDMD-N. GSDMD-N binds to the cell membrane and forms pores, facilitating the release of inflammatory cytokines and exacerbating the inflammatory response, ultimately contributing to the deterioration of ALI (57). In this study, the mRNA levels and protein expressions of NLRP3, ASC, GSDMD, CASP1, IL-18 and IL-1β were significantly reduced after treatment with QHSG, indicating that QHSG can inhibit the activation and assembly of NLRP3 inflammasome, as well as activation of CASP1. Furthermore, QHSG diminished the release of IL-18 and IL-1β, thereby preventing the aggravation of inflammation and ALI. Our findings are consistent with other documents that baicalin ameliorates hemorrhagic shock-induced lung injury in rats by inhibiting the activation of NLRP3 inflammasome via the NF-*κ*B pathway (58). In addition, amygdalin, another bioactive phytochemical of QHSG, is reported to possess a protective effect on LPS-induced ALI in mice by suppression of NF-*κ*B and NLRP3 signaling cascade (36). Collectively, the above evidences demonstrated that QHSG may alleviate the LPS-induced ALI by inhibition of NLRP3 inflammasome activation.

In the present study, we highlighted the regulatory effects of QHSG on TLR4/NF-*κ*B signaling pathway activity and NLRP3 inflammasome activation in ALI. However, efforts should be made to investigate the effect of QHSG on the crosstalk between TLR4/NF-*κ*B signaling cascade and NLRP3 inflammasome activation in ALI. To thoroughly understand the action mechanism of QHSG on ALI, the influence of QHSG on other inflammatory signaling pathways, such as p38 mitogen-activated protein kinase (MAPK), IL-6/Janus kinase 2 (JAK)/signal transducer and activator of transcription 3 (STAT3) and phosphoinositide 3-kinase (PI3K) (59), should be clarified in future work.

## 5 Conclusion

Taken together, QHSG may resolve acute lung injury, by alleviating the pathological features, reversing the alteration in white blood cell profile and impeding the production of inflammatory cytokines in LPS-induced ALI of the rats, and hindering the generation of inflammatory cytokines in LPS-stimulated mouse macrophages. The efficacy of QHSG on acute lung injury may be attributed to the anti-inflammation and immunoregulation of QHSG through attenuation of TLR4/NF-*κ*B signaling pathway activity and inhibition of NLRP3 inflammasome activation. Our findings provide a novel insight into the action mechanism of QHSG and lay a mechanistic foundation for therapeutic intervention in acute lung injury with QHSG in clinical practice.

## Data availability statement

The original contributions presented in the study are included in the article/supplementary material. Further inquiries can be directed to the corresponding authors.

## Ethics statement

Ethical approval was not required for the studies on humans in accordance with the local legislation and institutional requirements because only commercially available established cell lines were used. The animal study was approved by Animal Ethics Committee of Chengdu University of Traditional Chinese Medicine. The study was conducted in accordance with the local legislation and institutional requirements.

## Author contributions

ST: Conceptualization, Formal Analysis, Data curation, Investigation, Methodology, Writing – original draft. YL: Investigation, Methodology, Writing – review & editing. MW: Investigation, Methodology, Writing – review & editing. JL: Investigation, Methodology, Writing – review & editing. YP: Investigation, Methodology, Writing – review & editing. QT: Writing – review & editing, Data curation, Formal Analysis. TM: Data curation, Formal Analysis, Writing – review & editing. WY: Data curation, Formal Analysis, Writing – review & editing, Investigation, Methodology. CZ: Writing – review & editing, Conceptualization. JG: Conceptualization, Writing – review & editing, Funding acquisition, Project administration. HX: Conceptualization, Funding acquisition, Writing – review & editing, Formal Analysis, Supervision.
